# Supplementary figures and images for: Metagenomics Insight into Veterinary and Zoonotic Pathogens Identified in Urban Wetlands of Los Lagos, Chile
Source: Pathogens. 2024 Sep 12;13(9):788. doi: 10.3390/pathogens13090788 (PMC11434653; doi:10.3390/pathogens13090788)

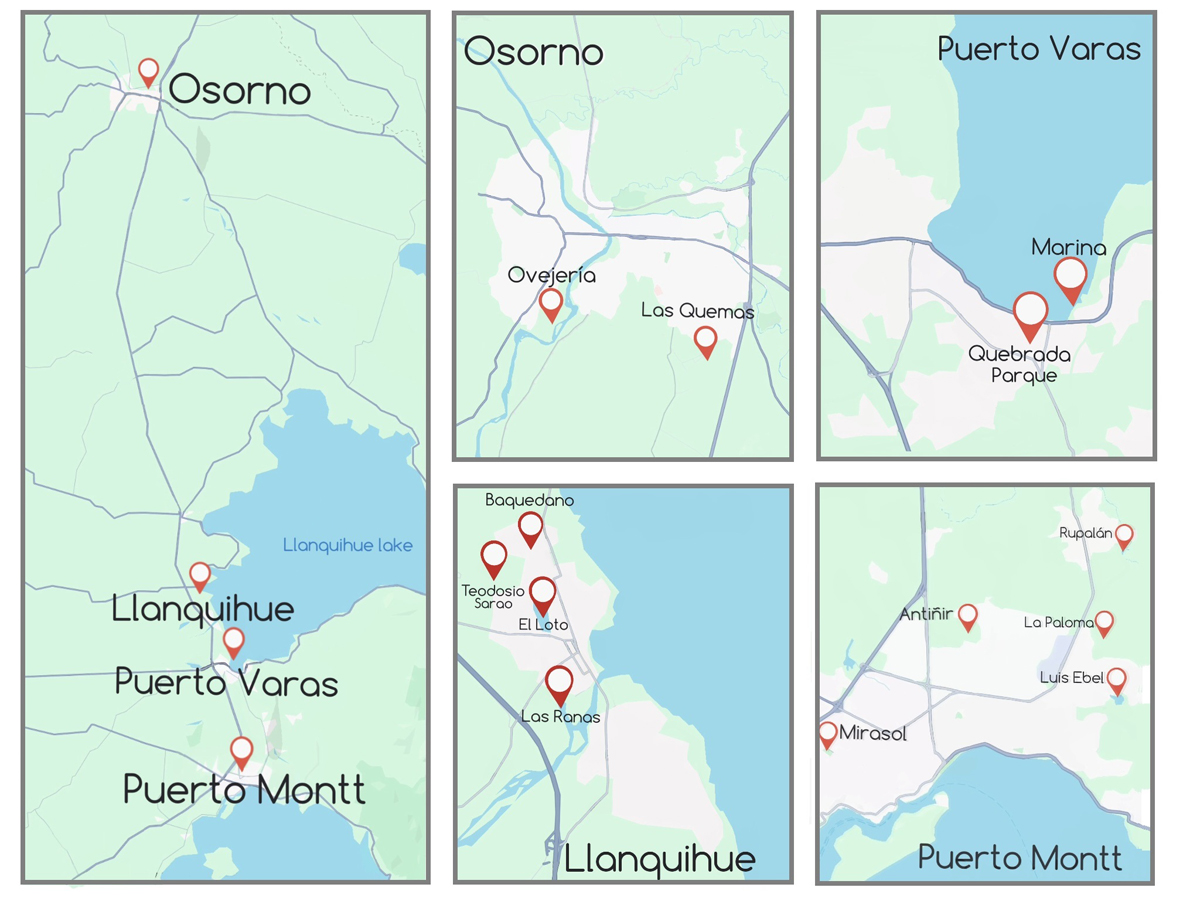

Supplement: Supplementary file 1 [file pathogens-13-00788-s001.zip › Supplementary Figure S1.jpg]

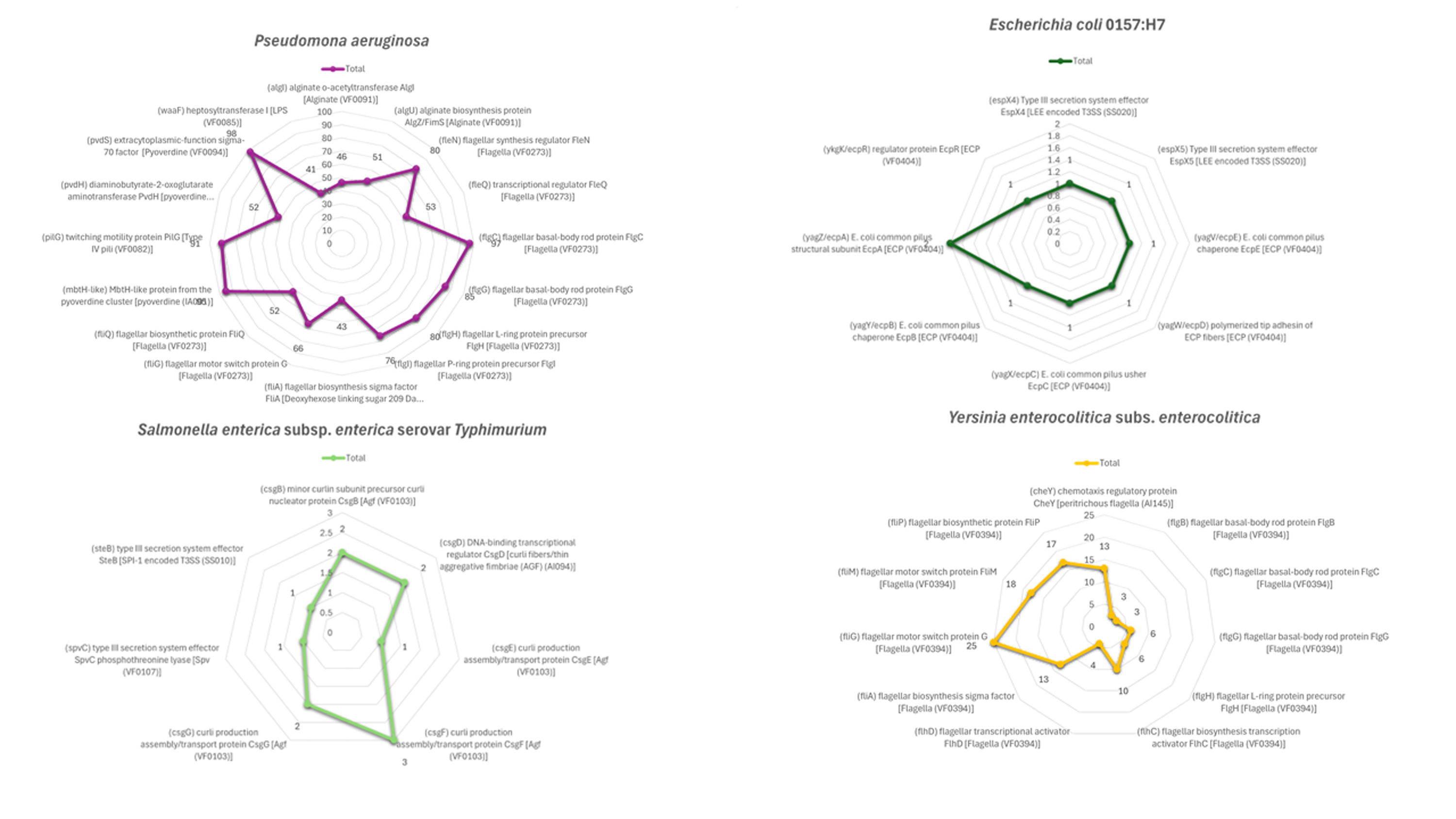

Supplement: Supplementary file 1 [file pathogens-13-00788-s001.zip › Supplementary Figure S2.png]
